# Supplementary material for: An interferon-integrated mucosal vaccine provides pan-sarbecovirus protection in small animal models
Source: Nat Commun. 2023 Oct 24;14:6762. doi: 10.1038/s41467-023-42349-5 (PMC10598001; doi:10.1038/s41467-023-42349-5)

Supplementary Information for:

An interferon-integrated mucosal vaccine provides pan-sarbecovirus protection in small animal models

Chun-Kit Yuen [a,b,#], Wan-Man Wong [a,b,#], Long-Fung Mak [a,b,#], Joy-Yan Lam [a,b], Lok-Yi Cheung [a], Derek Tsz-Yin Cheung [a], Yau-Yee Ng [a], Andrew Chak-Yiu Lee [a,b], Kwok-Yung Yuen [a, b, c, *], Kin-Hang Kok [a, b, c, d, *, †]

Author Affiliations

[a] Department of Microbiology, Li Ka Shing Faculty of Medicine, The University of Hong Kong, Hong Kong SAR, China.

[b] Centre for Virology, Vaccinology and Therapeutics, Hong Kong Science and Technology Park, Hong Kong SAR, China.

[c] State Key Laboratory for Emerging Infectious Diseases, The University of Hong Kong, Hong Kong SAR, China.

[d] AIDS Institute, Li Ka Shing Faculty of Medicine, The University of Hong Kong, Hong Kong SAR, China.

Author Notes

[#]Chun-Kit Yuen, Wan-Man Wong, and Long-Fung Mak contributed equally.

*Kwok-Yung Yuen and Kin-Hang Kok contributed equally.

[†] Corresponding to Kin-Hang Kok, khkok@hku.hk

This PDF file includes Supplementary Figure and Supplementary Figure Legend 1-14.

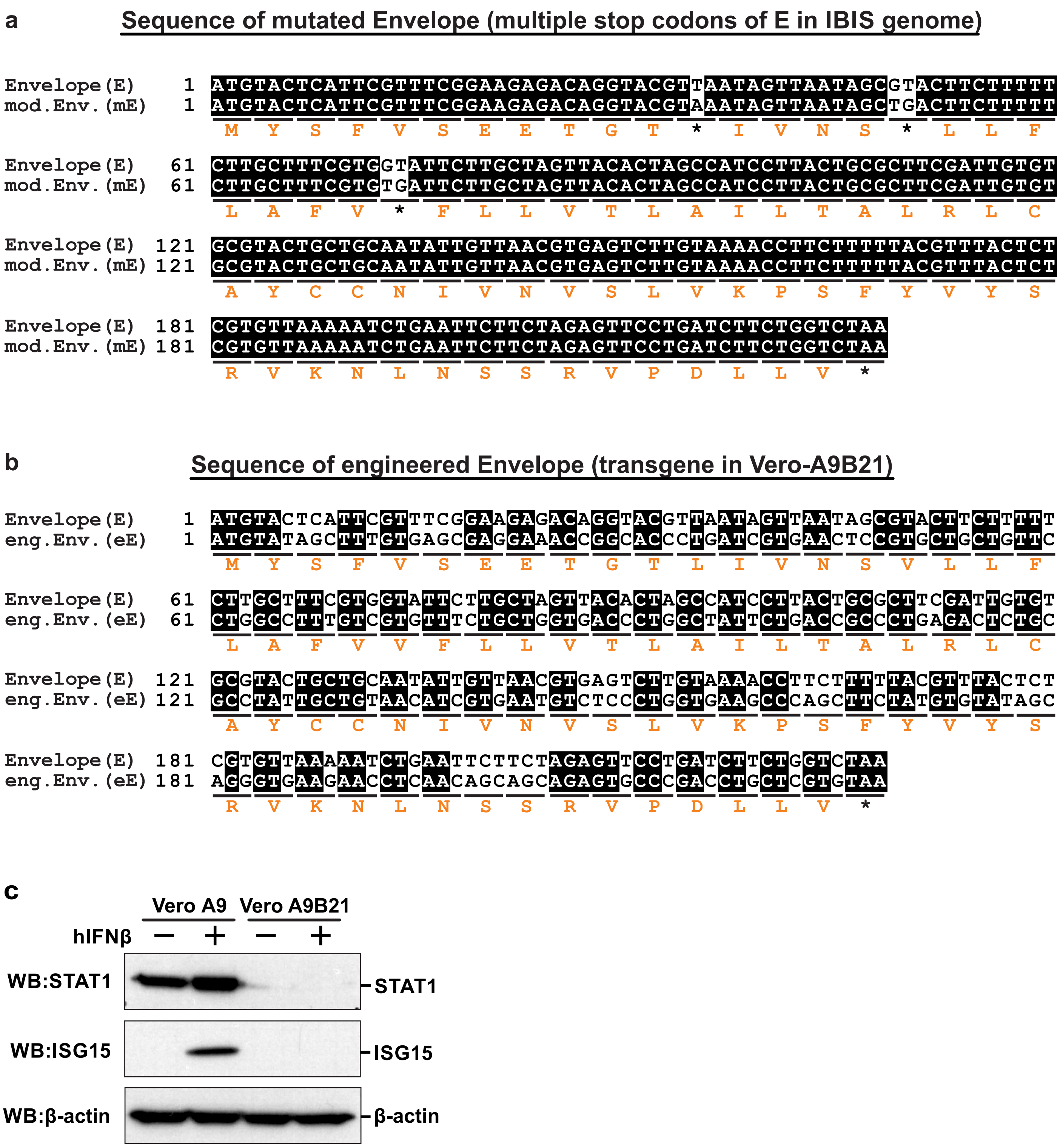

Supplementary Figure 1. Unique design of vaccine production cells. a Nucleotide sequence alignment between wildtype envelope (E) and the modified envelope (mE) in IBIS. Three early stop codons (indicated with *) were introduced into the envelope gene of IBIS to abolish the viral envelope protein expression. Black boxes and white boxes represent matched nucleotides and mismatched nucleotides respectively. Orange alphabets indicate amino acids translated by mE. **b** Nucleotide sequence alignment between wildtype envelope (E) and the engineered envelope (eE) transgene in the IBIS-production Vero A9B21 cells. Ninety synonymous nucleotide substitutions were introduced into the eE transgene. eE encodes the same amino acids as wildtype envelope. **c** STAT1 knockout in Vero A9B21 cells. After selecting the clonal Vero A9 cell stably expressing mE, the cell was further knocked out of the STAT1 gene. A single knockout clone Vero A9B21 was identified. Knockout of STAT1 was validated by western blotting. Ablation of interferon signaling was confirmed by the absence of ISG15 induction upon treatment with recombinant human IFNβ protein (1000 IU/mL) for 24 hours.

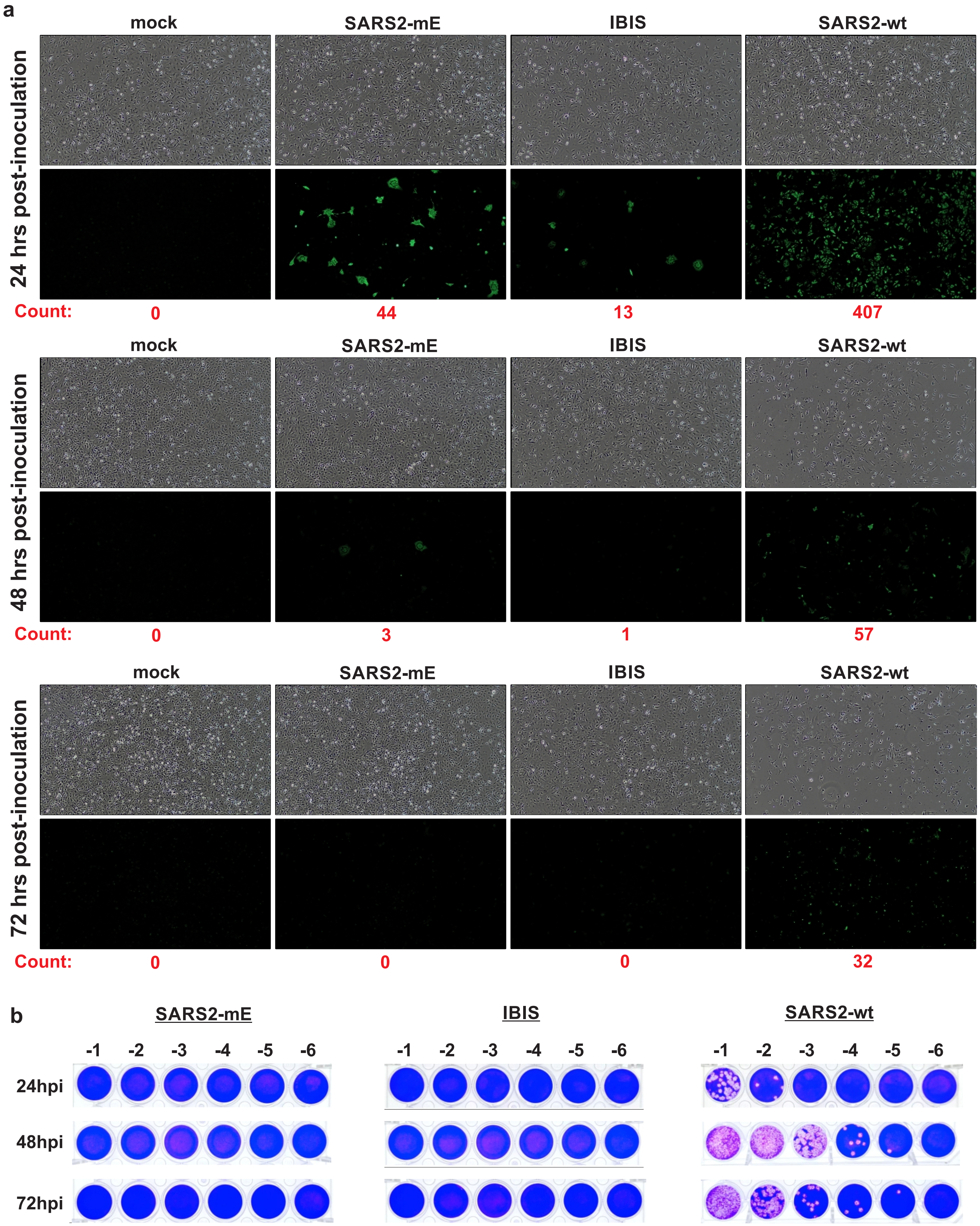

Supplementary Figure 2. Single-round infection of IBIS in L929-hACE2 cells. a Immunostaining of L929-hACE2. L929-hACE2 cells were either mock-infected, or infected with SARS2-mE, IBIS, or wildtype SARS-CoV-2 virus. Cells were PFA-fixed at 24, 48, and 72 hours post-infection, and stained with anti-SARS-CoV-2 N antibody. Cytopathic effect was only observed in brightfield images of wild-type SARS-CoV-2-infected cells at 48 and 72 hours post-infection. Identical images of cells harvested at 24 hours post-infection are shown in Fig. 1d. The number in red colour below each fluorescence image represents the number of green fluorescent foci counted using ImageJ software (v.1.54d). **b** Plaque assay of supernatant of infected L929-hACE2 in VeroE6 cells. Cell culture supernatant of the infected cells was harvested at 24, 48 and 72 hours post-infection. Amount of infectious virus was quantitated by plaque assay and the representative plaque assay images were shown. SARS2-wt, wildtype SARS-CoV-2; hpi, hours post-infection.

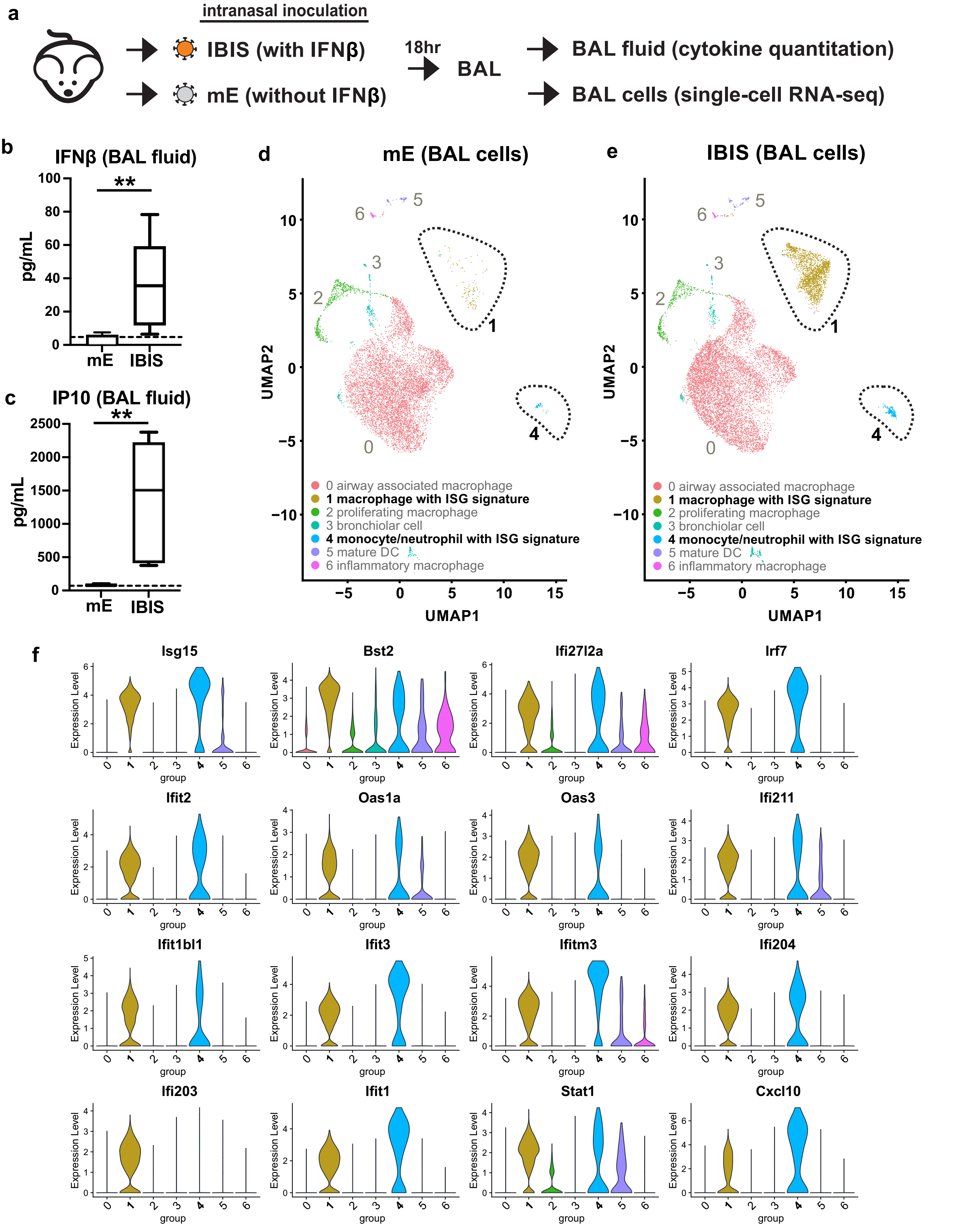

Supplementary Figure 3. Interferon induction and single-cell RNA sequencing of bronchoalveolar lavage cells harvested at 18 hours post-vaccination. a Schematic diagram showing the experimental setup. Six to ten week-old male K18-hACE2 transgenic mice were intranasally vaccinated with 1×10^5 PFU of SARS2-mE (mE) or IBIS (n=3 per group). At 18 hours post-vaccination, mice were sacrificed and bronchoalveolar lavage (BAL) was harvested. The BAL fluid was subjected to cytokine quantitation (**b-c**). BAL cells of each group were then pooled and subjected to single-cell RNA sequencing (scRNA-seq). **d-e** scRNA-seq clustering and UMAP reduction maps. Seven cell clusters (0-6) were identified and highlighted in different colours. Dotted lines indicate two cell populations with interferon-stimulated gene (ISG) signature. **f** Violin plots showing the expression of individual ISGs in the 7 cell-clusters. The UMAPs and violin plots were generated by R using ggplot2 function. Source data are provided as a Source Data file.

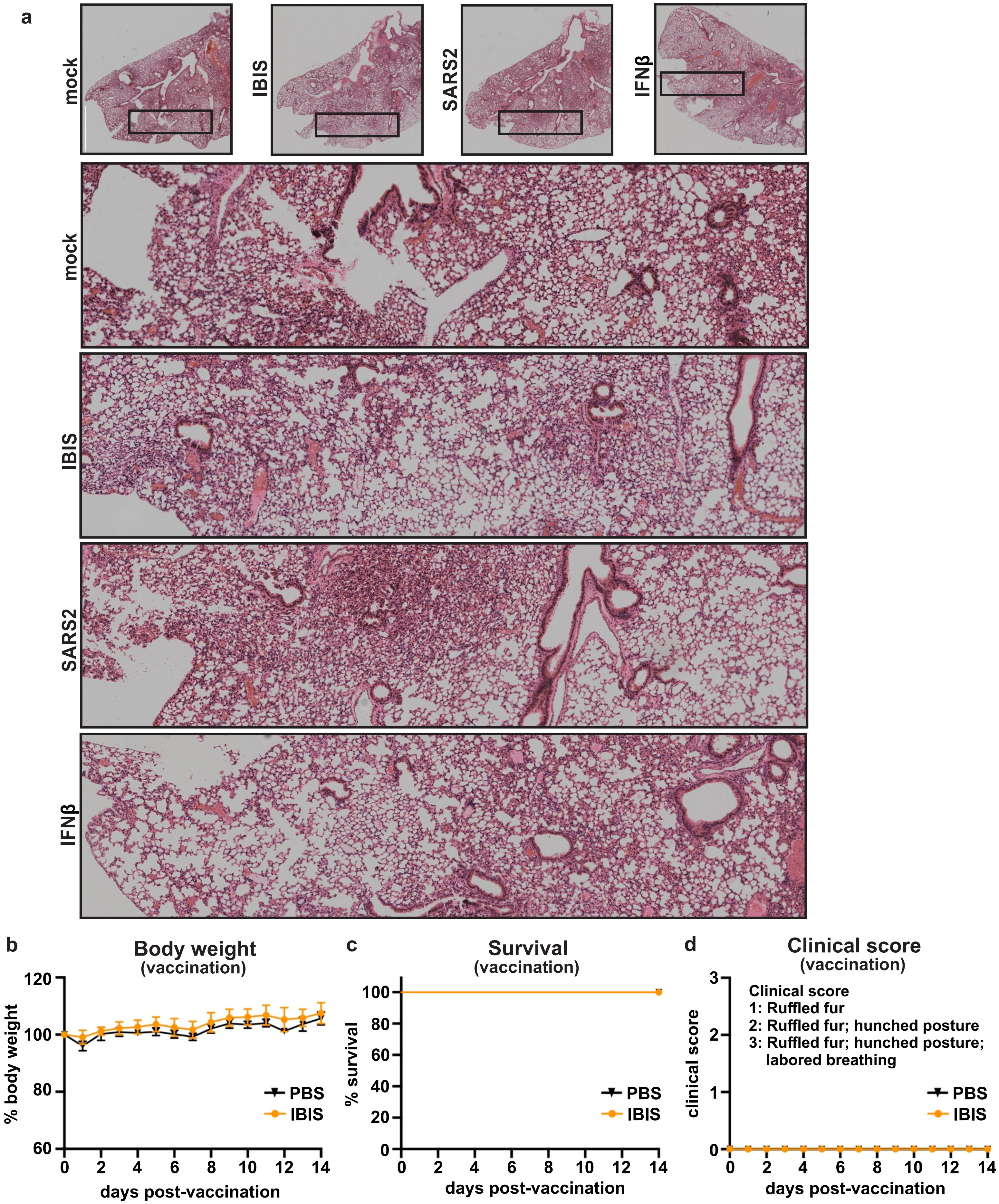

Supplementary Figure 4. Histology of IBIS-inoculated mouse lungs and health monitoring of mice for 14 days after IBIS vaccination.
a Images of hematoxylin and eosin (H&E)-stained mouse lung sections in Fig. 2h. K18-hACE2 transgenic mice were either mock-infected, or infected with IBIS (1×10^6 PFU) or SARS-CoV-2 (ancestral) (1×10^3 PFU), or treated with recombinant mouse IFNβ (1×10^5 IU). At 24 hours post-infection/treatment, the left lung was harvested and PFA-fixed for H&E staining. The upper panels show the brightfield images of the whole lung section captured with Cytation7 Imager (BioTek). Enlarged images of the selected regions are shown in lower panels. A portion of each enlarged image was cropped and shown in Fig. 2h. Six to ten week-old male K18-hACE2 transgenic mice were intranasally inoculated with either PBS (n=4) or IBIS (1×10^6 PFU) (n=5). Body weight (**b**), survival (**c**) and disease symptoms (**d**) of mice were monitored for 14 days. Source data are provided as a Source Data file.

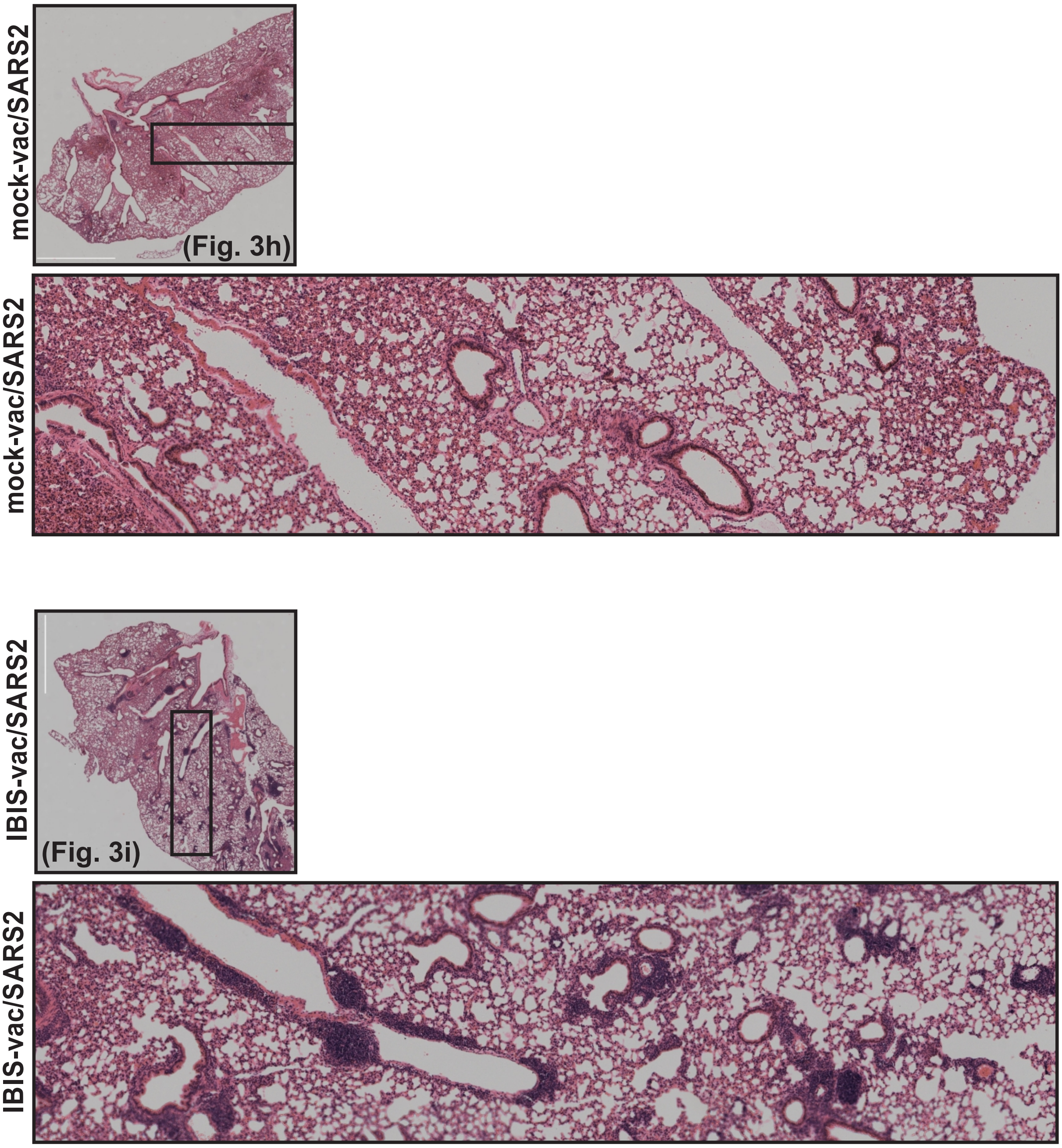

Supplementary Figure 5. Histology of IBIS-vaccinated mouse lungs at day 2 post SARS2-CoV-2 infection. Images of H&E-stained mouse lung sections in Fig. 3h and 3i. K18-hACE2 transgenic mice were either mock-vaccinated, or vaccinated with 2 doses of IBIS vaccine intranasally (1×10^{6} PFU/dose) at a 14-day interval (n=5 per group). At 14 days post-second dose of vaccination, mice were lethally challenged with SARS-CoV-2 (ancestral) (1×10^{3} PFU). At day 2 post-infection, the left lung was harvested and PFA-fixed for H&E staining. Enlarged images of the selected regions are shown in lower panels.

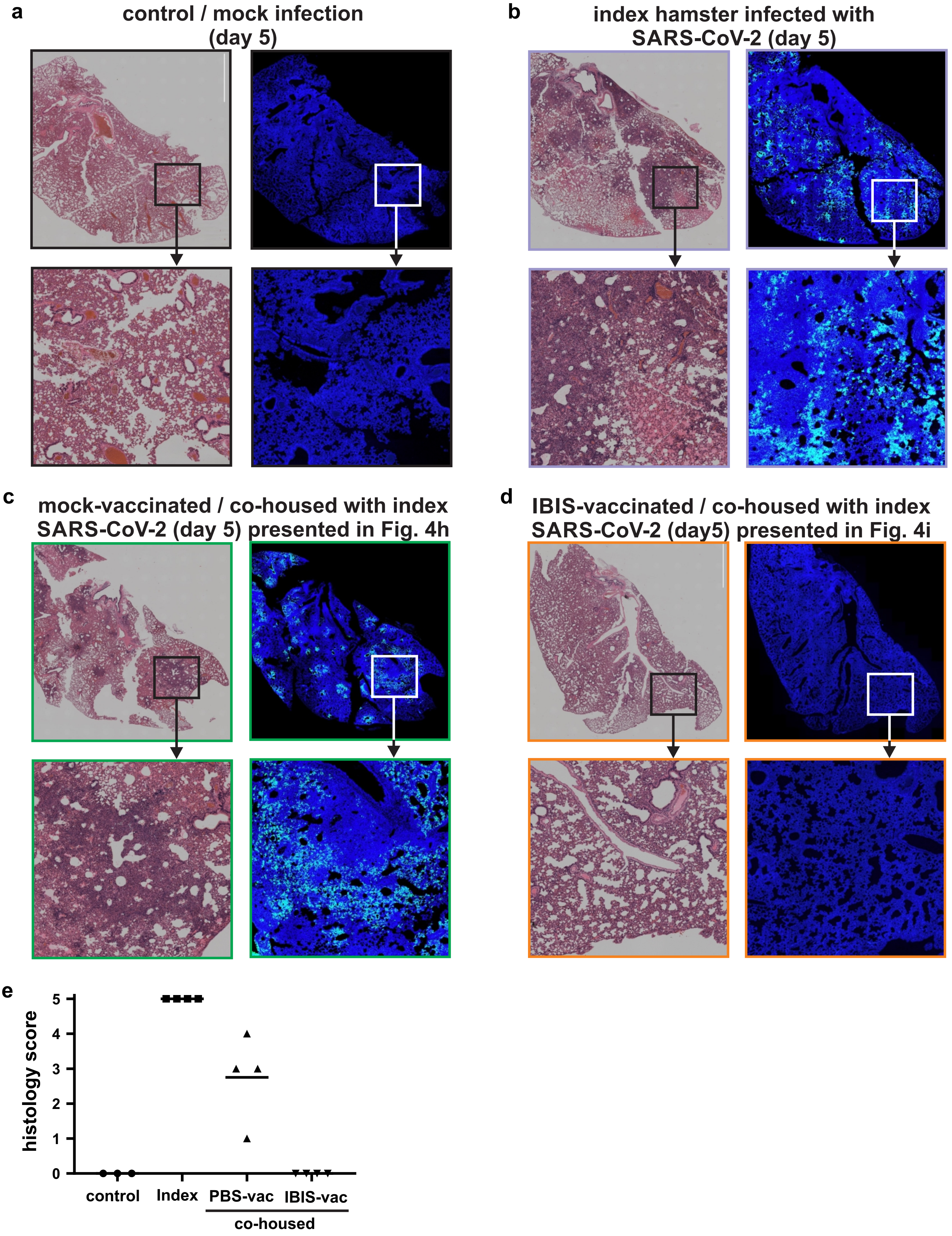

Supplementary Figure 6. Histology of SARS2-CoV-2 infected index/co-housed hamster lungs. Histology of hamster lungs from the experiment described in Fig. 4h-4i. Mock- or IBIS-vaccinated hamsters were co-housed with SARS-CoV-2-infected hamsters (index) for 1 day and then separated. Lungs of the hamsters were harvested at 5 days post-infection/co-housing. **a** mock-infected (n=3). **b** SARS-CoV-2 infected (index) (n=4). **c** PBS-vaccinated/co-housed (n=4). **d** IBIS-vaccinated/co-housed (n=4). Tissues were fixed in 4% PFA and paraffin-embedded. Tissue sections were then H&E stained or stained for SARS-CoV-2 N by immunostaining. **c** and **d** are also presented in the Fig. 4h and 4i respectively. **e** Histology scoring. The histopathology of hamster lungs was scored using the following criteria. 0, apparently normal; 1, mild mononuclear cell infiltration; 2, moderate mononuclear cell infiltration and alveolar thickening; 3, Oedema and <5% area of alveolar collapse; 4, 5%-20% area of alveolar collapse; 5, >20% area of alveolar collapse. Source data are provided as a Source Data file.

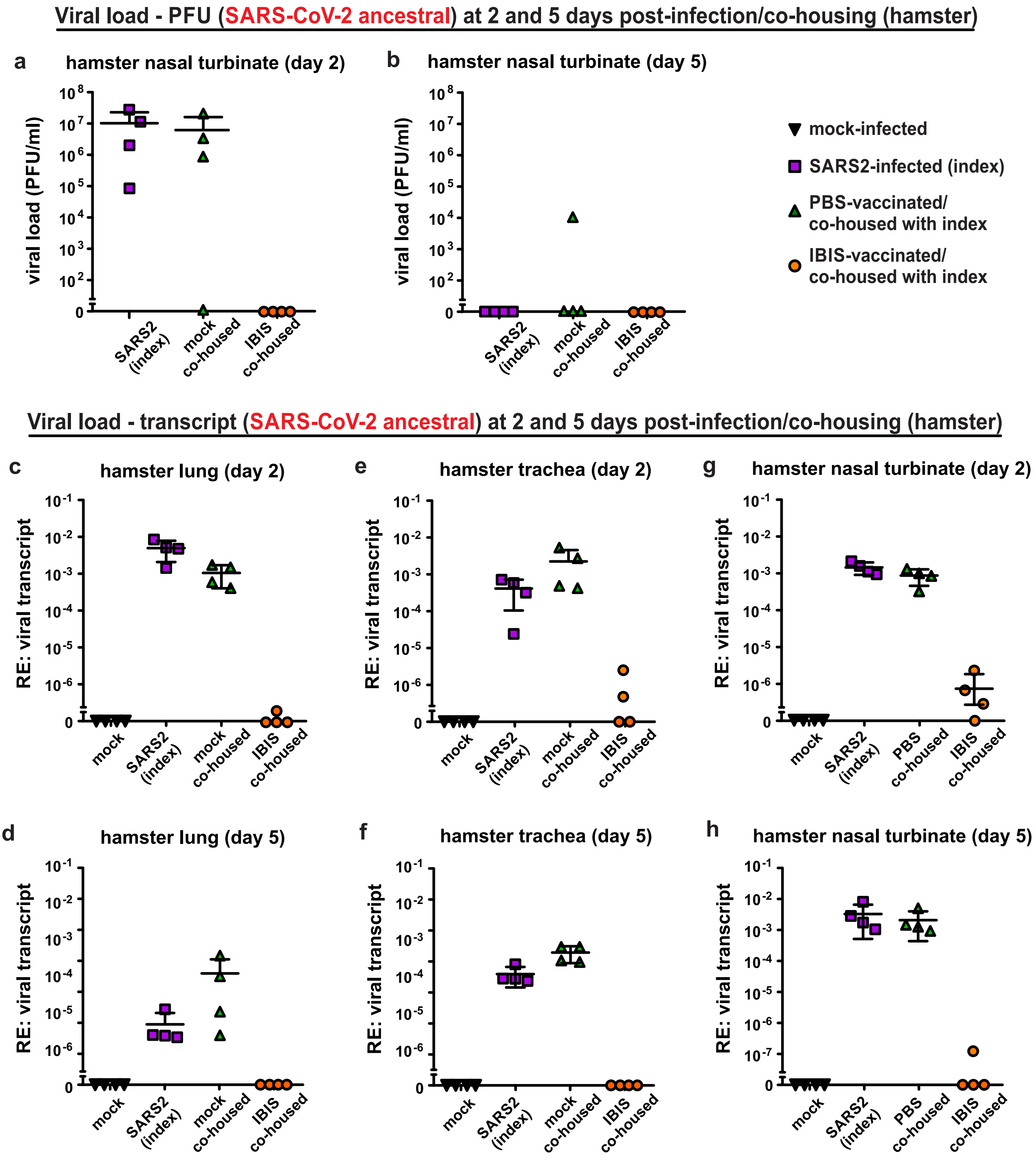

Supplementary Figure 7. Viral titer and transcript level in the respiratory tract tissues of SARS-CoV-2 infected/co-housed hamsters.
a-b Quantitation of viral titer in nasal turbinate of SARS-CoV-2 (ancestral) infected index/co-housed hamsters at day 2 and day 5 post infection/co-housing by plaque assay (n=4 per group), with the corresponding lung viral titer presented in Fig. 4f and 4g. Viral transcript level in the lungs (**c-d**), trachea (**e-f**) and nasal turbinate (**g-h**) of the hamsters were quantitated by RT-qPCR. RNA was extracted and reverse-transcribed into cDNA, which was then quantitated by SYBR Green qPCR and normalized to 18S rRNA. Source data are provided as a Source Data file.

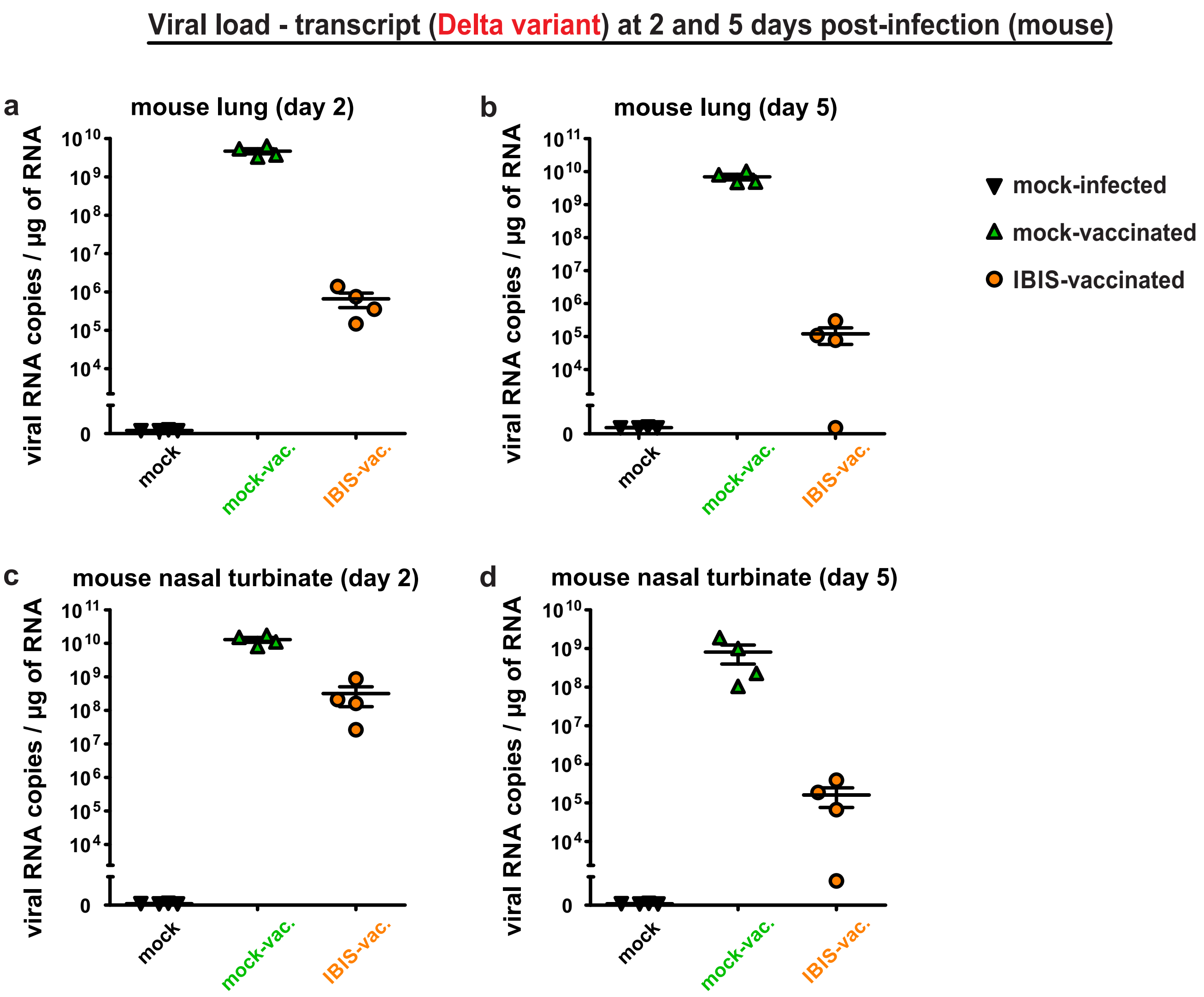

Supplementary Figure 8. Viral transcript level of SARS-CoV-2 (Delta) infected mice. Viral transcript level in the lungs (**a-b**) and nasal turbinate (**c-d**) of the mice infected with SARS-CoV-2 Delta variant, as described in Fig. 5a, was quantitated by one-step RT-qPCR using a TaqMan probe (n=4 per group). The transcript level was normalized to RNA input. Source data are provided as a Source Data file.

Viral load - transcript (Omicron-BA.1 variant) at 2 and 5 days post-infection (mouse)

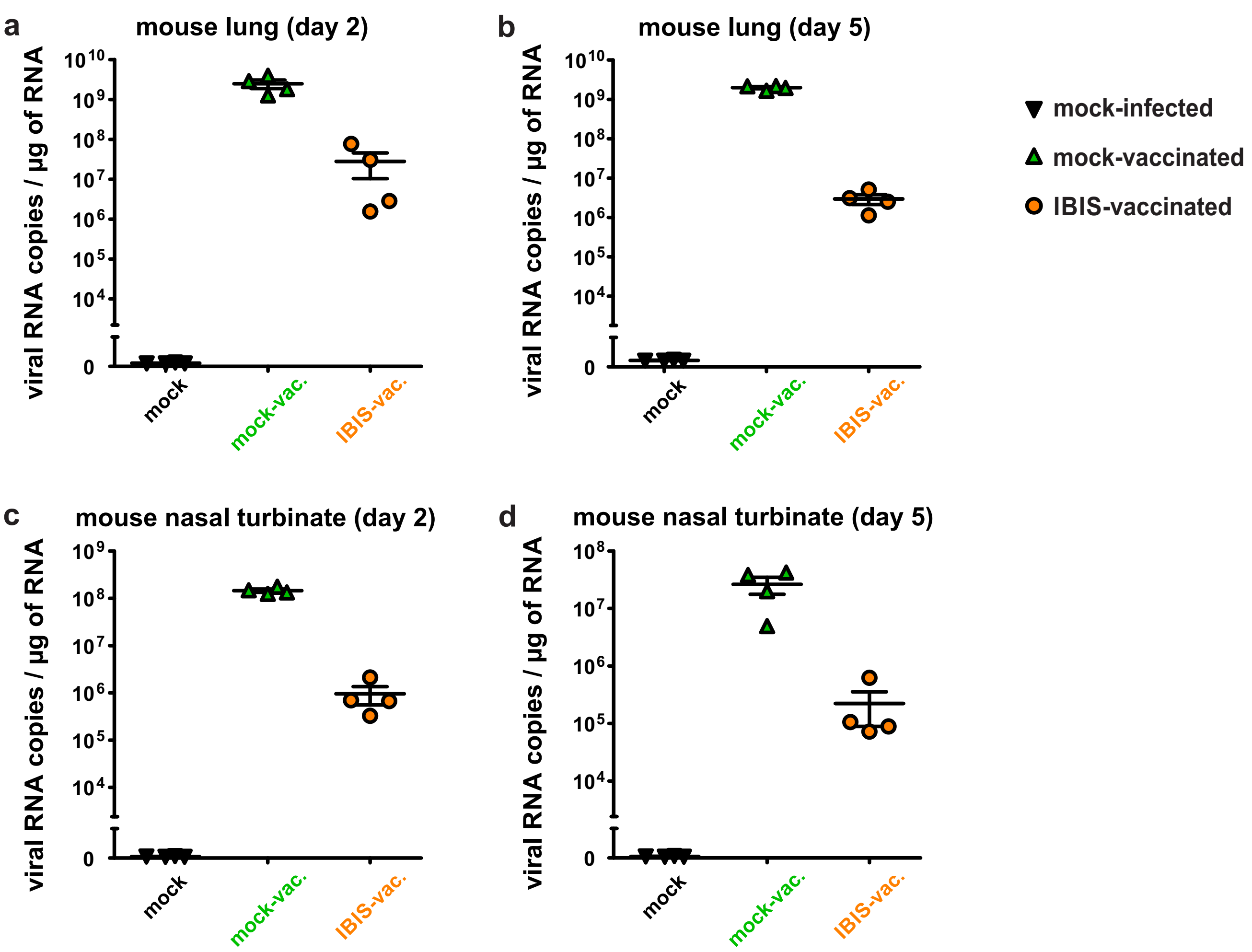

Supplementary Figure 9. Viral transcript level of SARS-CoV-2 (Omicron-BA.1) infected mice. Viral transcript level in the lungs (**a-b**) and nasal turbinate (**c-d**) of the mice infected with SARS-CoV-2 Omicron-BA.1 variant, as described in Fig. 5b, was quantitated by one-step RT-qPCR using a TaqMan probe (n=4 per group). The transcript level was normalized to RNA input. Source data are provided as a Source Data file.

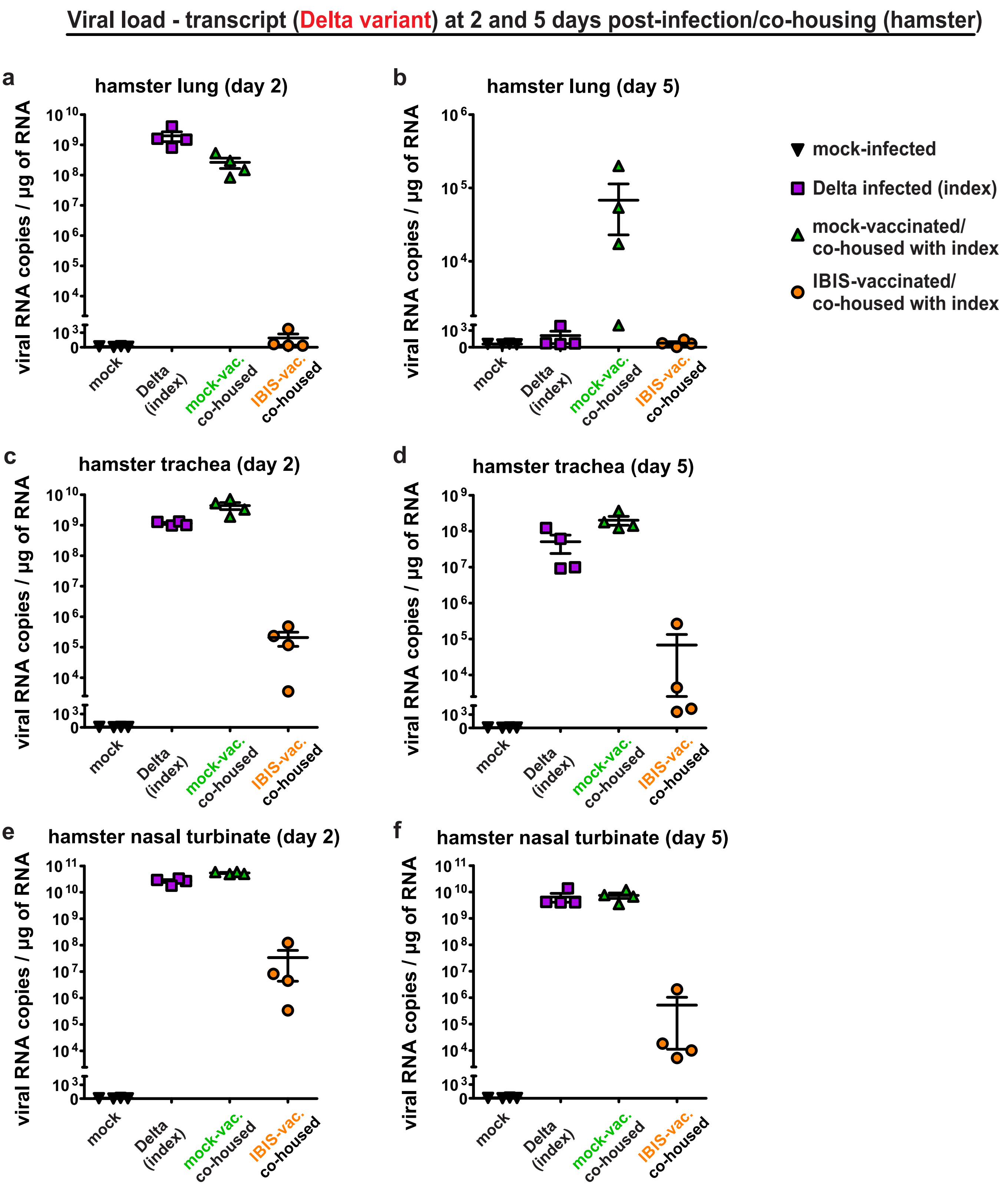

Supplementary Figure 10. Viral transcript level of SARS-CoV-2 (Delta) infected/co-housed hamsters. Viral transcript level in the lungs (**a-b**), trachea (**c-d**) and nasal turbinate (**e-f**) of the hamsters infected with SARS-CoV-2 Delta variant and the co-housed hamsters, as described in Fig. 5e, was quantitated by one-step RT-qPCR using a TaqMan probe (n=4 per group). The transcript level was normalized to RNA input. Source data are provided as a Source Data file.

Viral load - transcript (Omicron-BA.1 variant) at 2 and 5 days post-infection/co-housing (hamster)

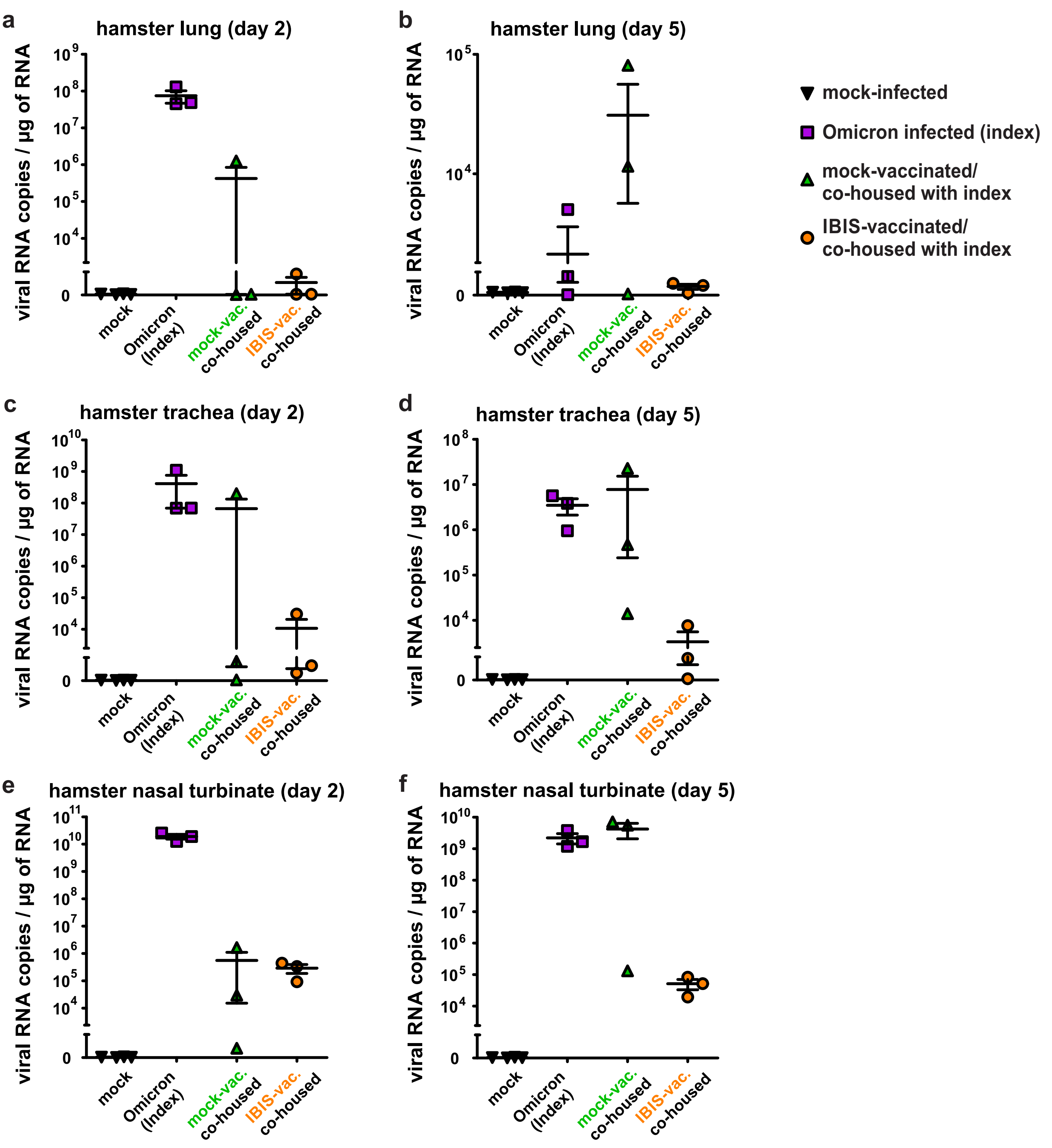

Supplementary Figure 11. Viral transcript level of SARS-CoV-2 (Omicron-BA.1) infected/co-housed hamsters. Viral transcript level in the lungs (**a-b**), trachea (**c-d**) and nasal turbinate (**e-f**) of the hamsters infected with SARS-CoV-2 Omicron-BA.1 variant and the co-housed hamsters, as described in Fig. 5g, was quantitated by one-step RT-qPCR using a TaqMan probe. Mock-infected (n=4). SARS-CoV-2 infected (index) (n=4). PBS-vaccinated/co-housed (n=4). IBIS-vaccinated/co-housed (n=3). The transcript level was normalized to RNA input. Source data are provided as a Source Data file.

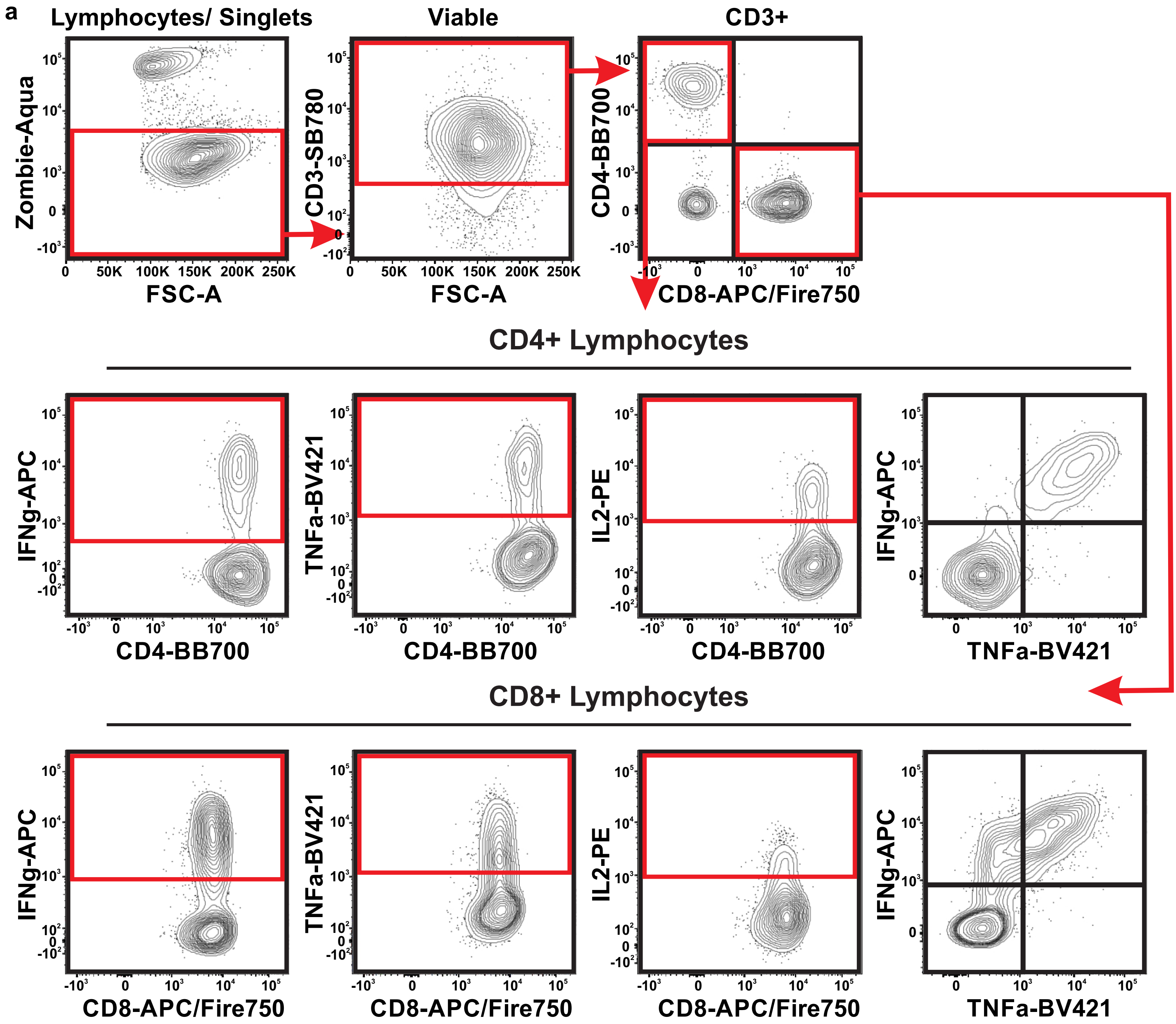

Supplementary Figure 12. Gating strategy for flow cytometric analysis. Fluorescence-activated cell sorting (FACS) profiles of bronchoalveolar lavage (BAL) cells showing the gating strategy applied for the quantification of antigen-specific activated CD4+ T cells and CD8+ T cells in mouse BAL, lung and spleen. Quadrant gates shown in FACS plots were set according to fluorescence-minus-one (FMO) controls using corresponding isotype-matched control antibodies.

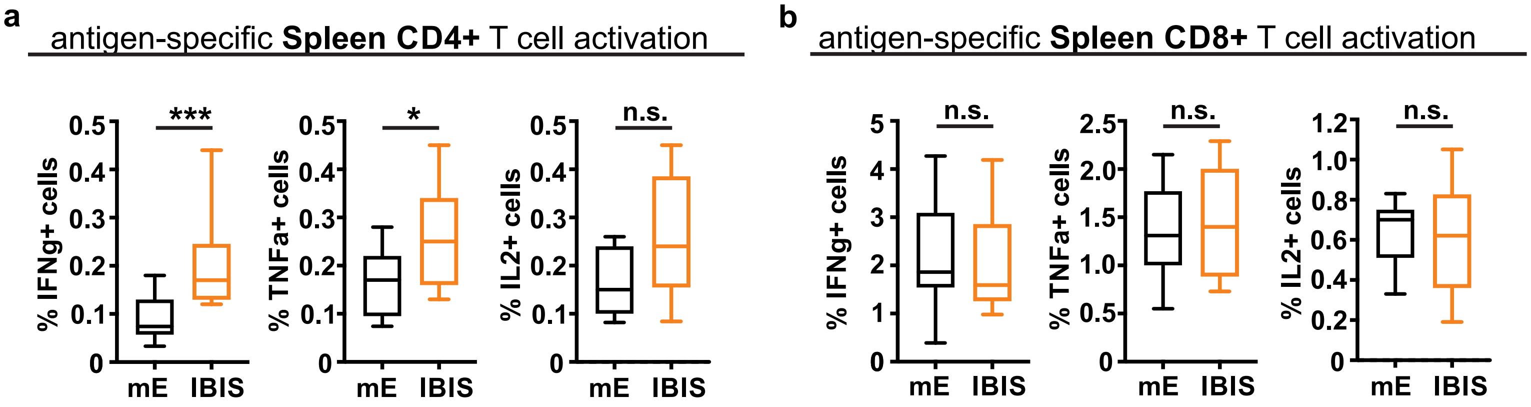

Supplementary Figure 13. Activation of antigen-specific T cells in spleen after IBIS or SARS2-mE vaccination. Splenic cells were stimulated with SARS-CoV-2 spike glycoprotein peptide pool followed by immunofluorescence staining. Cells stained for surface markers (CD3, CD4 and CD8) and intracellular cytokines (IFNγ, TNFα and IL2) were analyzed by flow cytometry. Activation of the antigen-specific CD4+ (**a**) or CD8+ (**b**) T cells were presented as a percentage of the total cell count of CD4+ or CD8+ T cells respectively. The results are from 4 independent experiments with the total number of mice per group: PBS (n=10). mE (n=11). IBIS (n=13). mE, SARS2-mE. Statistical analysis was performed using non-parametric Mann-Whitney test. ***, $p<0.001$; *, $p<0.05$; n.s., not significant. Source data are provided as a Source Data file.

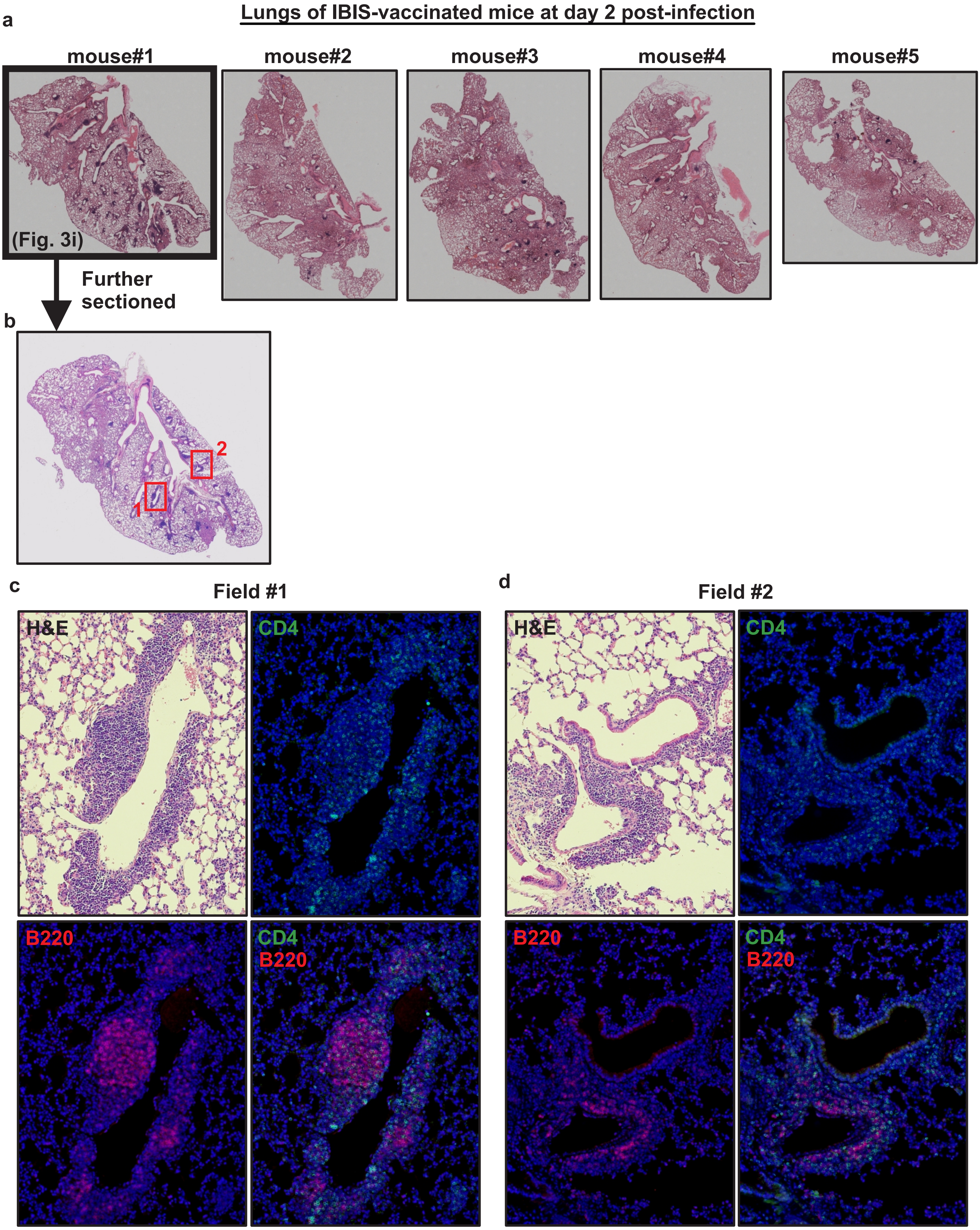

Supplementary Figure 14. H&E and immunofluorescence staining of IBIS-vaccinated mouse lungs at day 2 post SARS2-CoV-2 infection. **a** H&E staining of the lung sections of 5 IBIS-vaccinated mice infected with SARS-CoV-2 (ancestral), as exemplified in Fig. 3i and lower panels of Supplementary Fig. 5. The adjacent serial lung sections of the indicated mouse were further evaluated by H&E staining (**b**) and immunostaining for CD4 (green) and B220 (purple). Nuclei were counterstained by Hoechst 33258. Two selected fields on the H&E- and immuno-stained lung sections were enlarged and shown (**c-d**). Images were taken using a fluorescence microscope.

Uncropped western blotting images used in Supplementary Figure 1c

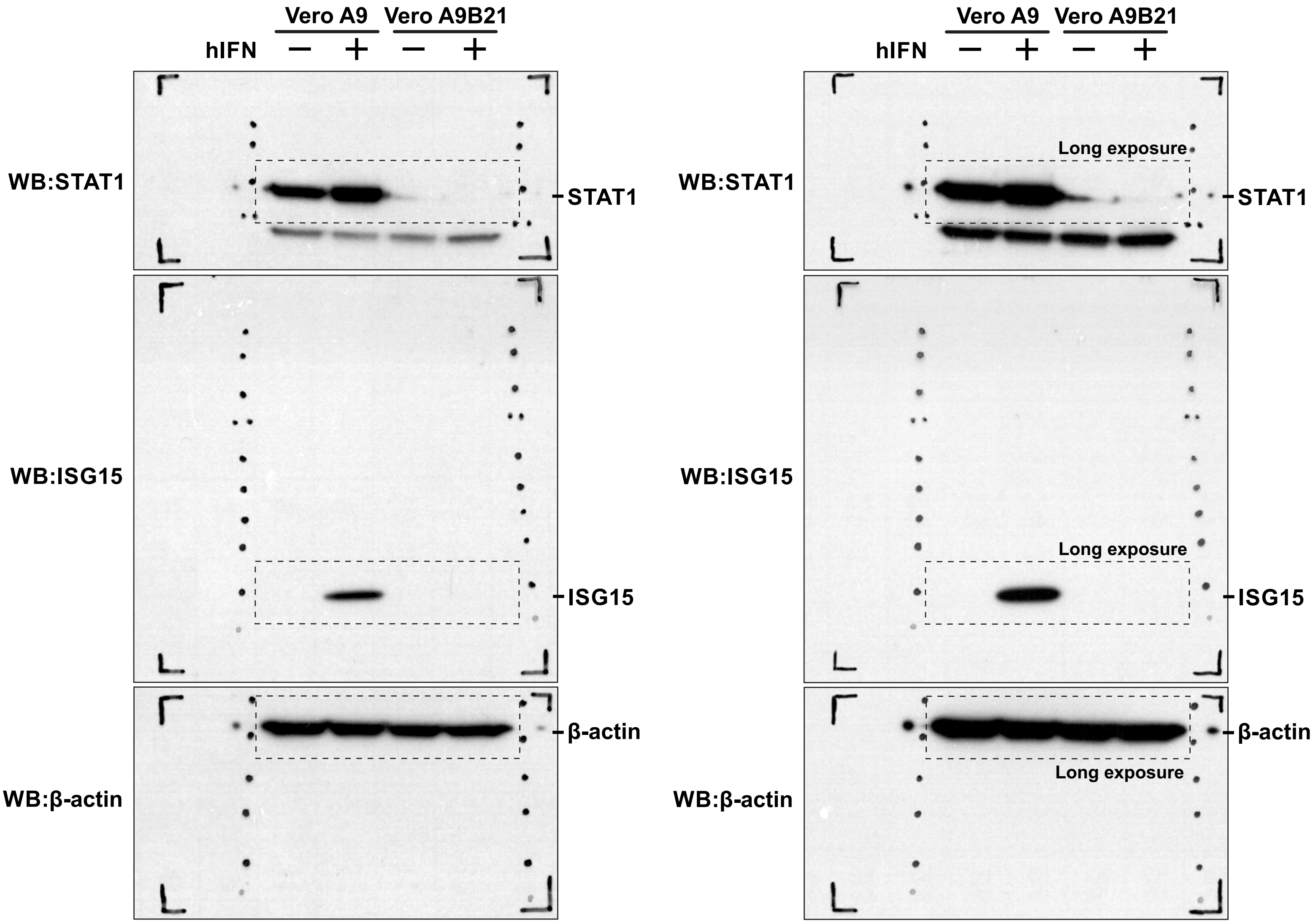

Supplement: Supplementary file 1 — Supplementary information [file 41467_2023_42349_MOESM1_ESM.pdf]
