# Supplementary material for: An interferon-integrated mucosal vaccine provides pan-sarbecovirus protection in small animal models
Source: Nat Commun. 2023 Oct 24;14:6762. doi: 10.1038/s41467-023-42349-5 (PMC10598001; doi:10.1038/s41467-023-42349-5)
Supplement: Supplementary file 3 — Reporting Summary [file 41467_2023_42349_MOESM3_ESM.pdf]

nature portfolio

Corresponding author(s): Kin-Hang Kok

Last updated by author(s): May 26, 2023

Reporting Summary

Nature Portfolio wishes to improve the reproducibility of the work that we publish. This form provides structure for consistency and transparency in reporting. For further information on Nature Portfolio policies, see our Editorial Policies and the Editorial Policy Checklist.

Statistics

For all statistical analyses, confirm that the following items are present in the figure legend, table legend, main text, or Methods section.

n/a	Confirmed	
☐	☒	The exact sample size (*n*) for each experimental group/condition, given as a discrete number and unit of measurement
☐	☒	A statement on whether measurements were taken from distinct samples or whether the same sample was measured repeatedly
☐	☒	The statistical test(s) used AND whether they are one- or two-sided<br>*Only common tests should be described solely by name; describe more complex techniques in the Methods section.*
☒	☐	A description of all covariates tested
☒	☐	A description of any assumptions or corrections, such as tests of normality and adjustment for multiple comparisons
☐	☒	A full description of the statistical parameters including central tendency (e.g. means) or other basic estimates (e.g. regression coefficient) AND variation (e.g. standard deviation) or associated estimates of uncertainty (e.g. confidence intervals)
☐	☒	For null hypothesis testing, the test statistic (e.g. *F*, *t*, *r*) with confidence intervals, effect sizes, degrees of freedom and *P* value noted<br>*Give P values as exact values whenever suitable.*
☒	☐	For Bayesian analysis, information on the choice of priors and Markov chain Monte Carlo settings
☒	☐	For hierarchical and complex designs, identification of the appropriate level for tests and full reporting of outcomes
☒	☐	Estimates of effect sizes (e.g. Cohen's *d*, Pearson's *r*), indicating how they were calculated

Our web collection on statistics for biologists contains articles on many of the points above.

Software and code

Policy information about availability of computer code

Data collection	Fluorescence foci was scanned by the Cytation 7 cell imaging muti-mode reader (BioTek).<br>Fluorescence images were captured with LSM900 confocal microscope (Zeiss).<br>Data of quantitative PCR were captured by ViiA7 (Thermo Fisher Scientific).<br>ELISA readings were measured by Varioskan LUX (Thermo Fisher Scientific).<br>Flow cytometry data were collected by LSRFortessa (BD Biosciences).
Data analysis	This study used commercially available Prism software v.8.0 and v.9.5.0 (GraphPad) for data analysis, including the data presentation and statistical analysis.<br>The number of foci was counted with Gen5 software (BioTek).<br>Fluorescence images were processed by ZEN version 3.7 (Zeiss).<br>Figures were prepared using CorelDraw software (2020).<br>Quantitative PCR data were processed using QuantStudio Real-Time PCR software version 1.3 (Thermo Fisher Scientific) and Excel software (Microsoft).<br>ELISA readings were processed using Skanlt Software version 6.0.1 (Thermo Fisher Scientific) and the corresponding end-point titers were calculated using Excel software (Microsoft) and Prism software v.8.0 (GraphPad).<br>Flow cytometry data were analysed using FlowJo version 10 (BD Biosciences).

For manuscripts utilizing custom algorithms or software that are central to the research but not yet described in published literature, software must be made available to editors and reviewers. We strongly encourage code deposition in a community repository (e.g. GitHub). See the Nature Portfolio guidelines for submitting code & software for further information.

Data

Policy information about availability of data

All manuscripts must include a data availability statement. This statement should provide the following information, where applicable:

- Accession codes, unique identifiers, or web links for publicly available datasets
- A description of any restrictions on data availability
- For clinical datasets or third party data, please ensure that the statement adheres to our policy

The complete sequence of SARS-CoV-2 HKU-001a (GenBank: MT230904), B.1.617.2/Delta variant (GenBank: OM212471) B.1.1.529/Omicron variants BA.1 (GenBank: OM212472), BA.2 (GISAID: EPI_ISL_9845731), BA.4.1 (GISAID: EPI_ISL_13777657), BA.5.2 (GISAID: EPI_ISL_13777658) and SARS-CoV-1 GZ50 (GenBank: AY304495) are available on GenBank or GISAID.
Single-cell RNA sequencing data have been deposited onto NCBI Sequence Read Archive (BioProject: PRJNA1002063) (https://www.ncbi.nlm.nih.gov/bioproject/PRJNA1002063).

Research involving human participants, their data, or biological material

Policy information about studies with human participants or human data. See also policy information about sex, gender (identity/presentation), and sexual orientation and race, ethnicity and racism.

Reporting on sex and gender	n/a
Reporting on race, ethnicity, or other socially relevant groupings	n/a
Population characteristics	n/a
Recruitment	n/a
Ethics oversight	n/a

Note that full information on the approval of the study protocol must also be provided in the manuscript.

Field-specific reporting

Please select the one below that is the best fit for your research. If you are not sure, read the appropriate sections before making your selection.

[x] Life sciences [ ] Behavioural & social sciences [ ] Ecological, evolutionary & environmental sciences

For a reference copy of the document with all sections, see nature.com/documents/nr-reporting-summary-flat.pdf

Life sciences study design

All studies must disclose on these points even when the disclosure is negative.

Sample size	No sample size calculation was performed. Samples sizes for mouse and hamster models were determined based on our previous published results obtained in in-vivo experiments with similar settings (PMID: 35062016 and 33727703).
Data exclusions	Outliers were identified using ROUT method (GraphPad Prism version 9.5.0). Neither the presence nor absence of the outliers would change the statistically significant difference between the datasets. Excluded data points are shown in the source data file of Figure 7 as crossed out. ROUT method is pre-established and widely used, as referenced in<br>Motulsky HM and Brown RE, Detecting outliers when fitting data with nonlinear regression – a new method based on robust nonlinear regression and the false discovery rate, BMC Bioinformatics 2006, 7:123.
Replication	All experiments were performed in at least two independent biological replicates. Similar findings were obtained from all repeats.
Randomization	Sex- and age matched mice were randomly assigned to each mock and vaccination group (with or without infections).
Blinding	Investigators were not blinded for the following analyses (pathogenesis, mortality, collection of viral shedding samples) due to the biosafety concerns associated with sample handling in BSL3 laboratory. For the antibody titers, viral load and cytokine quantitation, experimenters were blinded. A unique sample ID was given to each sample and passed to the experimenters for quantitation without knowing the identity of samples.

Reporting for specific materials, systems and methods

We require information from authors about some types of materials, experimental systems and methods used in many studies. Here, indicate whether each material, system or method listed is relevant to your study. If you are not sure if a list item applies to your research, read the appropriate section before selecting a response.

Materials & experimental systems

n/a	Involved in the study
☐	☒ Antibodies
☐	☒ Eukaryotic cell lines
☒	☐ Palaeontology and archaeology
☐	☒ Animals and other organisms
☒	☐ Clinical data
☒	☐ Dual use research of concern
☒	☐ Plants

Methods

n/a	Involved in the study
☒	☐ ChIP-seq
☐	☒ Flow cytometry
☒	☐ MRI-based neuroimaging

Antibodies

Antibodies used	Anti-mouse CD8a-APC-Fire750 (Rat IgG2ak; 0.2mg/mL; Biolegend 100766) 1:400<br>Anti-mouse CD3-SB780 (Rat IgG2bk; 0.2mg/mL; eBioscience 78-0032-82) 1:100<br>Anti-mouse IL2-PE (Rat IgG2bk; 0.2mg/mL; Biolegend 503808) 1:100<br>Anti-mouse TNFα-BV421 (Rat IgG1k; 0.2mg/mL; Biolegend 506328) 1:200<br>Anti-mouse IFNγ-APC (Rat IgG1k; 0.2mg/mL; Biolegend 505809) 1:100<br>Anti-mouse CD107a-AF488 (Rat IgG2ak; 0.5mg/mL; eBioscience 53-1071-82) 1:400<br>Anti-mouse Granzyme B-PECy7 (Rat IgG2ak; 0.2mg/mL; eBioscience 25-8898-82) 1:100<br>Anti-mouse CD4-BB700 (Rat IgG2ak; 0.2mg/mL; BD 566408) 1:400<br>Anti-mouse CD4 (Rabbit IgG; 0.687 mg/ml; Abcam ab183685) for immunofluoresence at 1:2000<br>Anti-mouse B220-APC (Rat IgG2ak; 0.2mg/mL; Biolegend 103212) for immunofluoresence at 1:100<br>In-house rabbit anti-SARS-CoV-2 nucleoprotein (N) immune serum for immunofluorescence at 1:1000<br>Goat anti-Rabbit IgG (H+L), Alexa Fluor 488 (A-11034, Thermo Fisher Scientific) for immunofluoresence at 1:500<br>Goat anti-Rabbit IgG (H+L), Alexa Fluor 488 (A-11008, Thermo Fisher Scientific) for immunofluoresence at 1:500
Validation	Antibodies were titrated and validated prior to the experiments. The specificity and application of commercial primary antibodies were validated by the manufacturers and validation statements are available on the manufacturers' website. The in-house anti-SARS-CoV-2 N immune serum was validated and reported in our previous publications (Nat Commun. 2021 12:134 and Nature 2022 609:785-792)

Eukaryotic cell lines

Policy information about cell lines and Sex and Gender in Research

Cell line source(s)	The following commercially available cell-lines were used in this study:<br>Vero E6 (ATCC, CRL-1586), VeroE6/TMPRSS2 (JCRB Cell Bank, JCRB1819), L929 (ATCC, CCL-1), BHK-21 (ATCC, CCL-10) and A549-hACE2-hTMPRSS2 (Invivogen, a549-hace2tpsa).
Authentication	Morphology for the cell-line was assessed by microscopy. Permissiveness of the cell-line was assessed through the observation of cytopathic effect by microscopy and quantitation of virus produced by plaque forming assay.
Mycoplasma contamination	All cell-lines were tested negative for mycoplasma.
Commonly misidentified lines<br>(See ICLAC register)	No commonly misidentified cell lines were used in this study.

Animals and other research organisms

Policy information about studies involving animals; ARRIVE guidelines recommended for reporting animal research, and Sex and Gender in Research

Laboratory animals	K18-hACE2 transgenic mice (B6.Cg-Tg(K18-ACE2)2Prlmn/J; source: The Jackson Laboratory) and Golden Syrian Hamsters (Mesocricetus auratus; source: Envigo, USA) were used in our study. The animals were housed and bred under an AAALAC International accredited program at the Centre for Comparative Medicine Research, HKU.<br>K18-hACE2 mice, male and female, aged from 6-10 weeks were used. Golden Syrian hamster, female, aged from 6-10 weeks were used. Animals were kept in open or individually ventilated cages under a 12:12 dark light cycle within environmentally controlled rooms (65% humidity and ambient temperature ranging between 21-23oC). Animals were fed ad libitum with laboratory diet manufactured by LabDiet, USA.
Wild animals	No wild animals were used in this study.
Reporting on sex	Both male and female K18-hACE2 mice were used in this study. Either sex was used in each experiment, depending on the animal availability at CCMR, HKU. Female hamsters were used in this study for consistency. No preference on sex has been taken since both sexes were susceptible to SARS-CoV-2 infections.

Field-collected samples	No field-collected samples were used in this study.
Ethics oversight	All animal experiments were performed with prior approval from the Committee on the Use of Live Animals in Teaching and Research (CULATR), The University of Hong Kong and under licence from the Hong Kong SAR Government's Department of Health. Animal infection experiments were performed in the Animal Biosafety Level 3 (BSL3) laboratory of The University of Hong Kong, in accordance with the Block T Animal Facility Safety Regulations Manual and Standard Operating Procedures (SOP-V220200101) and approved CULATR protocols.

Note that full information on the approval of the study protocol must also be provided in the manuscript.

Flow Cytometry

Plots

Confirm that:

- [x] The axis labels state the marker and fluorochrome used (e.g. CD4-FITC).
- [x] The axis scales are clearly visible. Include numbers along axes only for bottom left plot of group (a 'group' is an analysis of identical markers).
- [x] All plots are contour plots with outliers or pseudocolor plots.
- [x] A numerical value for number of cells or percentage (with statistics) is provided.

Methodology

Sample preparation

<Lung, splenic and BAL cell preparation>
1. Sacrifice mice with overdose isoflurane.
2. Perform the transcardial perfusion by injecting 10mL PBS/1% SP into the right ventricle (with the incision at the posterior vena cava near the kidney).
3. Harvest BAL in 2mL of PBS/5% FBS/1%SP and transfer to sterile 1.5mL microcentrifuge tube. Keep on ice.
4. Transfer the whole lung into the well of sterile 60mm dish containing PBS/1% PS for rinsing away blood. Then transfer the tissue to 5mL tube containing 2mL tissue digestion medium. Keep on ice.
5. Transfer the spleen directly into 5mL tube containing 2mL tissue digestion medium. Keep on ice.
<Lung>
6. Transfer the lung into a new 60mm petri-dish with no medium/ PBS inside.
7. Cut the lung into pieces by scissors.
8. Add 2mL of Tissue Digestion Medium into the petri dish. Examine the lung tissues and cut into smaller pieces if necessary. Keep on ice until all lung samples are processed.
9. Add 1mL digestion solution into each petri dish. Mix well by rotation. Incubate the dish at 37ºC for 30 minutes. Spin the dish in circular motion every 10 minutes.
10. After 30 minutes, add 30µL 0.5M EDTA solution into each dish to stop the digestion. Mix by rotation. Keep on ice for 10 minutes.
11. Assemble 100µm filter onto 50mL falcon. Pre-wet the filter with Tissue Digestion Medium.
12. Use 1mL plastic Pasteur pipette to transfer the lung pieces and the digestion solution onto the central area of the filter. Remove the cells from the bottom of the dish as well.
13. Use the black side of the plastic plunger from 3mL syringe for grinding. Pour Tissue Digestion Medium onto the filter for 3-4 times during grinding.
14. Spin down cells at 1500rpm for 5 minutes at room temperature.
15. Pour off the supernatant. Loosen the cell pellet by hitting the 50mL falcon tube by hand.
16. Add 2mL 1X cell lysis buffer into each tube. Spin the tube in circular motion for most of the time and incubate for 1 minute.
17. Add PBS/1% SP up to 20mL in the tube to stop lysis.
18. Spin down cells at 1500rpm for 5 minutes at room temperature.
19. Pour off the supernatant. Loosen the cell pellet by hitting the 50mL falcon tube by hand
20. Add 10mL PBS/1% PS into each tube.
21. Spin down cells at 1500rpm for 5 minutes at room temperature.
22. Pour off the supernatant.
23. Resuspend the cells in 0.85mL T-cell activation medium.
24. Count cells using machine.
25. Prepare cell suspension at the density of 2X10^6 cells/100µL T-cell activation medium.
<Spleen>
26. Assemble 70µm filter onto 50mL falcon. Pre-wet the filter with Tissue Digestion Medium.
27. Transfer the whole spleen and the remaining medium onto the filter.
28. Use the black side of the plastic plunger from 3mL syringe for grinding.
29. Pour Tissue Digestion Medium onto the filter for 3-4 times during grinding. The final volume would be 10-15 mL.
30. Spin down cells at 1500rpm for 5 minutes at room temperature.
31. Pour off the supernatant. Loosen the cell pellet by hitting the 50mL falcon tube by hand.
32. Add 3mL 1X cell lysis buffer into each tube. Spin the tube in circular motion for most of the time and incubate for 1 minute 20 seconds.
33. Add PBS/1% SP up to 20mL in the tube to stop lysis.

34. Spin down cells at 1500rpm for 5 minutes at room temperature.
35. Pour off the supernatant. Loosen the cell pellet by hitting the 50mL falcon tube by hand
36. Add 10mL PBS/1% PS into each tube.
37. Spin down cells at 1500rpm for 5 minutes at room temperature.
38. Pour off the supernatant. Loosen the cell pellet by hitting the 50mL falcon tube by hand.
39. Resuspend the cells in 3mL T-cell activation medium.
40. Filter the cells through 70µm filter into a new 50mL conical tube.
41. Count cells using machine.
42. Prepare cell suspension at the density of 2X10^6 cells/100µL T-cell activation medium.
<BAL>
43. Spin down the tubes containing BAL cells at 4000 rpm for 5 minutes.
44. Remove supernatant. Resuspend cells from the same samples (2 tubes/ sample) with 120µL T-cell activation medium.
45. Transfer 12µL of cell suspension into a new tube for manual cell counting.
46. Transfer 100µL of each sample into individual wells for culture.
47. Prepare the 100µL T-cell stimulation master mix containing Spike peptide pool (PepMix™ SARS-CoV-2 Spike Glycoprotein; JPT PM-WCPV-S-1) and plate correspondingly. Incubate the cells at 37ºC with 5% CO2. Add Brefeldin A and monensin, at a final concentration of 7µg/mL and 2µM repsectively, to the culture after 2-hour incubation and further incubate the cells for overnight.
<Antibody staining>
48. After overnight of incubation, prepare PBS/20mM EDTA.
49 Add 20µL of PBS/20mM EDTA into each well to halt the activation using multi-channel pipette. Mix well by pipetting up and down.
50. Incubate for 15 minutes at room temperature. Mix again by vigorous pipetting to fully resuspend adhered cells. Transfer the resuspension into a new 96-well V-bottom plate for staining.
51. Spin down plate at 500g for 5 minutes. Remove supernatant by flicking the plate. Add 250µL PBS into each well.
52. Spin down plate at 500g for 5 minutes. Remove supernatant by flicking the plate.
53. Resuspend cells in each well with 50µL Fc-blockade (1:200 in PBS).
54. Incubate at 4ºC for 10 minutes with shaking.
55. After 10-minute incubation, add 50µL antibody solution into the corresponding wells. Incubate at 4ºC for 15 minutes with shaking.
56. Add 150µL PBS/5% FCS into each well. Spin down cells at 500g for 5 minutes. Remove supernatant by flicking the plate.
57. Add 250µL PBS/5% FCS into each tube. Spin down cells at 500g for 5 minutes. Remove supernatant by flicking the plate
58. Vortex the plate briefly prior to the addition of Fixation/ Permeabilization solution so as to minimize cell aggregation.
59. Add 100µL Fixation/ Permeabilization solution into each well. Incubate for 20 minutes at 4ºC with shaking.
60. After 20-minute fixation/ permeabilization, add 150µL Perm/Wash buffer to the well, spin down cells at 800g for 5 minutes. Remove supernatant by flicking plate.
61. Add 250µL Perm/Wash buffer into each well. Spin down cells at 800g for 5 minutes. Remove supernatant by flicking the plate.
62. Resuspend cells with 50µL antibody solution prepared in Perm/wash buffer. Incubate at 4ºC for one hour with shaking.
70. Add 150µL Perm/wash buffer into each well. Spin down cells at 800g for 5 minutes. Remove supernatant by flicking the plate.
71. Add 250µL Perm/wash buffer into each well. Spin down cells at 800g for 5 minutes. Remove supernatant by flicking the plate.
72. Resuspend cells with 130µL PBS/5% FBS. Keep at 4ºC in dark with parafilm until FACS analysis.
73. Filter through 70µm cell strainer before FACS acquisition

Instrument: Flow cytometry data were collected by LSRFortessa (BD Biosciences).

Software: Flow cytometry data were analysed using FlowJo version 10 (BD Biosciences).

Cell population abundance: Upon request, a figure exemplifying the cell populations will be provided in the Supplementary Information

Gating strategy: A figure exemplifying the gating strategy is provided in the Supplementary Information

☒ Tick this box to confirm that a figure exemplifying the gating strategy is provided in the Supplementary Information.
